# The American Medical Association, Denver Meeting, June 7-10, 1898

**Published:** 1898-05

**Authors:** J. W. Graham

**Affiliations:** Chairman Committee of Arrangements; Denver, Colo.


### The American Medical Association, Denver Meeting, June 7-10, 1898.

DENVER, COLO., April 14, 1898.

Railroad Rates. The Western Passenger Association has granted a rate to Denver and return of one-half fare, plus $2.00, thirty-day limit, for business from Chicago, St. Louis and intermediate points. Tickets on sale June 2nd, 4th and 5th east of the Missouri River; 5th and 6th west of the Missouri River. A round trip rate of $20.00, thirty-day limit, from Ogden and Salt Lake, is also announced. Application for similar rates has been made to all other Passenger Associations and to rail-roads not controlled by them. Announcement of other rates and rules governing the sale of tickets will be made in *The Journal of the Association* as soon as decisions are received. This rate is as low as granted any convention this year.

J. W. GRAHAM, M. D.,
Chairman Committee of Arrangements.

